# *Ralstonia solanacearum* and *Xanthomonas perforans* as Causal Agents of Bacterial Disease of Tomato

**DOI:** 10.1002/mbo3.70195

**Published:** 2025-12-09

**Authors:** Mateka Patience Modiba, Thomas Bell, Bernard Glick, Olubukola Oluranti Babalola

**Affiliations:** ^1^ Food Security and Safety Focus Area, Faculty of Natural and Agricultural Sciences North‐West University Mmabatho South Africa; ^2^ Department of Life Sciences Imperial College London Berkshire UK; ^3^ Department of Biology University of Waterloo Waterloo Ontario Canada

**Keywords:** biological control agents, disease management, omics, *Ralstonia*, tomato disease, virulence factors, *Xanthomonas*

## Abstract

Tomatoes are produced worldwide, and in South Africa, they are cultivated in all provinces. The most destructive tomato diseases are bacterial spot, caused by *Xanthomonas* spp., and bacterial wilt caused by *Ralstonia solanacearum*. Over the years, different strategies have been employed to control tomato disease. The disadvantage of chemical pesticides is that they alter microbial communities and sometimes remain on food commodities. Recently, studies have been conducted on biological control agents in the hope of eventually replacing the use of chemical pesticides. Some studies have discovered potential biological control agents for bacterial diseases. Better insight into host‐pathogen interaction will help develop better disease management strategies. This review provides insights into plant diseases caused by *Ralstonia* and *Xanthomonas* and how they are managed.

## Graphical abstract

An extensive review of *Xanthomonas perforans* and *Ralstonia solanacearum*, two important tomato pathogens, with a focus on disease progression and new methods of management.

**Abbreviations**
ASM: acibenzolar‐s‐methyl
BCA: biological control agents
EPSs: exopolysaccharides
IPM: integrated pest management
ISR: induce systemic resistance
PAMPs: pathogen‐associated molecular patterns
PGPR: plant growth‐promoting rhizobacteria
PRRs: plant pattern recognition receptors
Rips: *Ralstonia*‐injected proteins
SAR: systematic acquired resistance
T1SS: type 1 secretion system
T2SS: type 2 secretion system
T3E: type 3 effectors
T3SS: type 3 secretion system
T4P: type 4 pili
T4SS: type 4 secretion system
T5SS: type 5 secretion system
T6SS: type 6 secretion system
TALE: transcription activator‐like effectors

## 1 Introduction

Tomatoes (*Solanum lycopersicum*) are one of the most commonly grown and consumed vegetables in the world (Bihon et al. 2022). Tomatoes are inexpensive and contain a variety of nutrients, including vitamins A, B, B1, B6, C, K, niacin, proteins, lipids, carbs, iron, and lycopene. Tomatoes are not only delicious but also nutritious (Ali et al. 2020). They play a significant role in health. For example, lycopene is an antioxidant that has protective benefits against several types of cancer and heart disease, among other benefits (Direk and Topkara 2018). Tomatoes are the second most grown plant in the world, but their production, both fresh and processed, is hindered by a variety of diseases caused by bacteria, fungi, viruses, viroids, and phytoplasmas (Ma et al. 2023). Cultivated tomatoes have a low genetic diversity due to extensive selection and severe genetic bottlenecks, making them more susceptible to disease. Tomatoes are affected by approximately 200 diseases caused by numerous pathogens during cultivation and postharvest around the world (Panno et al. 2021).

The most common tomato diseases are bacterial wilt disease caused by *Ralstonia solanacearum* strains, and bacterial spot caused by *Xanthomonas* spp (Morcia et al. 2023). The severity of these diseases increases under humid conditions, which results in devastating losses (Jibrin et al. 2022). Due to global warming increasing the atmospheric humidity, the increasing world population, and disease threats, there is a dire need to find solutions to protect and sustain the tomato industry (Singh et al. 2023). The various approaches to managing diseases fall into four categories: physical, chemical, cultural, and biological. These will be covered in more detail below. The most used approach is using chemicals; however, the use of chemicals harms plants as well as the environment, and farmers are looking into moving away from chemical usage (Baker et al. 2020).

The commonly proposed method is the utilization of biological control agents (BCA). The term biological control means using living organisms as “natural enemies” to eliminate or suppress plant diseases (Stenberg et al. 2021). These natural enemies are usually microorganisms that use different approaches to reduce the population density of pathogens. For instance, biological control agents can compete for space and organic nutrients, which will ultimately reduce the survival of the pathogen due to nutrient deprivation (Lahlali et al. 2022). They may also induce the plant's defense mechanism, which will result in induced systemic resistance of the plant, making it resistant to various pathogens (Collinge et al. 2022). Various putative BCAs have been suggested as being capable of managing plant diseases; however, little is known about the mechanisms they use (Lahlali et al. 2022). Omics‐based technologies can play a significant role in identifying or offering greater insights into the mode of action used by BCA, as well as identifying genes that are critical to making a BCA successful.

Tomato bacterial wilt and bacterial spot, which are caused by these pathogens, continue to be among the most destructive diseases limiting tomato production globally despite intensive research efforts. Their broad host range, high genetic variability, and environmental tolerance continue to pose challenges to current management approaches. Omics‐based technologies have emerged as powerful tools to unravel the complex molecular interactions underlying pathogenesis and host defense, given the growing demand for eco‐friendly and sustainable disease control (Kumar et al. 2025). Finding new targets for resistance breeding and biocontrol development is made easier by combining genomics, transcriptomics, proteomics, and metabolomic information to provide a deeper understanding of pathogen biology (Jain et al. 2024). This review gives an overview of bacterial infections caused by *Ralstonia solanacearum* and *Xanthomonas perforans*, as well as the most frequent disease management options. Furthermore, it highlights existing knowledge gaps, examines the potential of omics‐driven innovation in creating sustainable management strategies for tomato bacterial disease, and outlines future research directions crucial for converting molecular discoveries into useful field applications. Therefore, this review highlights existing knowledge gaps, examines the potential of omics‐driven innovations in creating sustainable management strategies for tomato bacterial diseases, and outlines future research directions crucial for converting molecular discoveries into useful field applications.

## 2 Virulence Strategies of Bacterial Pathogens of Tomato

Tomatoes are a South American crop that is widely grown around the world. Tomatoes are becoming increasingly popular as a staple in people's diets. Tomatoes are high‐yielding, adaptable, and nutritious. However, like with any other crop, tomatoes are susceptible to a variety of diseases, which have a significant impact on their yield and quality (Liu and Wang 2020). Fungi, bacteria, oomycetes, viruses, and nematodes are among the most common causative agents. Some diseases that affect tomatoes include bacterial spots, late light, leaf mold, yellow leaf curl virus, and gray leaf spot (Zhang et al. 2022).

**Figure 1:**
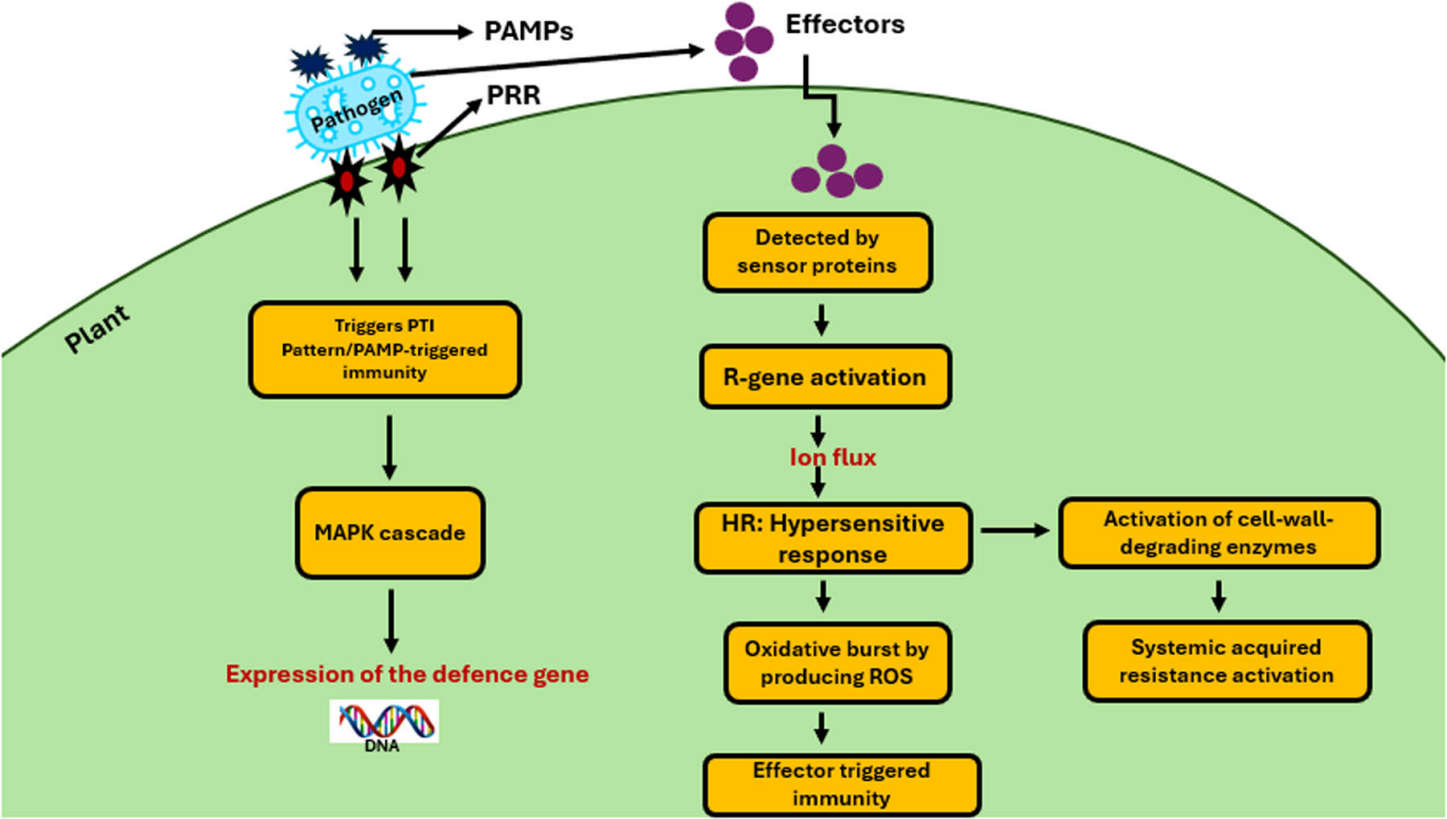
Overview of key molecular components involved in plant–pathogen interactions. The diagram illustrates the relationship between pathogen‐associated molecular patterns (PAMPs), pattern recognition receptors (PRRs), elicitors, effectors, and virulence factors, highlighting their roles in triggering plant immune responses and pathogen strategies to overcome host defenses.

Plants protect themselves against pathogens by secreting a variety of enzymes and antimicrobial elicitors. Pathogens secrete molecules to aid infection, but some are detected by plant pattern recognition receptors (PRRs), triggering the plant's immune responses. These immune‐activating molecules from either the pathogen or the plant are known as elicitors (Kaur et al. 2022; Li et al. 2023). Plants have pattern recognition receptors (PRRs), a type of receptor that may recognize plant pathogens and pathogen‐associated molecular patterns (PAMPs) (Figure 1). Pathogens also produce compounds that determine their pathogenicity and allow them to establish themselves within the host plant (Li and Wu 2021). These compounds have varied effects on the plant's health. Plant diseases can create problems such as utilizing the host plant's contents, lowering chlorophyll levels, reducing leaf area, interfering with the transfer of water and minerals, and interfering with the flow of phytohormones and vital enzymes. As previously indicated, these compounds change the plant's entire physiology, favoring disease infestation, expansion, and colonization (Singh et al. 2023; Nazarov et al. 2020).

These compounds are commonly known as microbial virulence factors, which help in overcoming the host defense system and spreading disease (Liu et al. 2022). This term includes cell surface features like capsules, glycol‐ and lipoproteins, lipopolysaccharides, and toxins, as well as released products like enzymes and exopolysaccharides (Abdalla et al. 2021). It is also known that intracellular changes in metabolic regulatory networks, which are regulated by protein sensors/regulators and non‐coding regulatory RNAs, affect virulence (Leitão 2020; Carezzano et al. 2023).

The type III secretion system is essential to many pathogenic Gram‐negative bacteria (T3SS). By injecting bacterial effector proteins into host cells, this system disrupts the host's defense mechanisms (Yuan et al. 2020). The *hrp* gene encodes this mechanism in bacterial plant pathogens, and it participates in eliciting the hypersensitive response (HR) and pathogenicity. The HR is a programmed cell death reaction that occurs locally in plants in response to pathogen identification at the site of infection (Puigvert et al. 2019).

Proteolysis is another strategy used by bacteria, which is the breakdown of proteins/peptides into amino acids, which is critical for infection. By breaking down the host's physical barriers, the released proteases allow for easier penetration and dissemination (Figaj et al. 2019). Furthermore, they facilitate host colonization by interfering with the host's defense mechanisms. These enzymes also serve a regulatory role, allowing the pathogen to initiate infection at the optimal time during environmental changes (Figaj et al. 2019).

The plant cell wall is a complex structure that serves as the first line of defense against microorganisms. Although plant cell walls have evolved to be resistant to mechanical damage and enzymatic deconstruction, pathogens continue to find a way through the barrier. Furthermore, phytopathogens produce cell‐wall‐degrading enzymes that are crucial for pathogenicity. These enzymes aid in the destruction, penetration, and diffusion of host tissue while also encouraging nutrition leakage from the host (Soria et al. 2022).

These virulence strategies illustrate bacterial pathogens' remarkable adaptability to tomato hosts and environments. While many mechanisms are shared across genera and species, the specific content, regulation, and expression of virulence determinants change significantly between pathogens. Understanding these pathogen‐specific differences is crucial for unraveling their distinct infection processes and establishing effective control strategies. The following sections, therefore, focus on the major virulence factors associated with *Ralstonia* and *Xanthomonas*, two important bacterial pathogens of tomato.

## 3 An Overview of *Ralstonia* as a Phytopathogen

*Ralstonia solanacearum* is a Gram‐negative bacterial soil‐borne pathogen responsible for the devastating bacterial wilt. It is known to be versatile and able to adapt rapidly to changing environments as well as to new hosts (Xia et al. 2023). Bacterial wilt caused by the *R. solanacearum* species complex causes diseases of major economic importance, impacting the production of various hosts, which include tomato, sunflower, banana, groundnuts, eggplant, pepper, potato, and ginger, amongst others. *R. solanacearum* is an extensively studied plant pathogen due to its tremendous destructive potential (Phiri et al. 2024). The pathogen is mostly found in hot and humid regions, while reports of its virulence have also been made in lower temperatures. *Ralstonia* populations can reach 10^3^–10^8^ cfu/g of soil and plant tissue, respectively, under heavy infection (Paudel et al. 2020).

**Figure 2:**
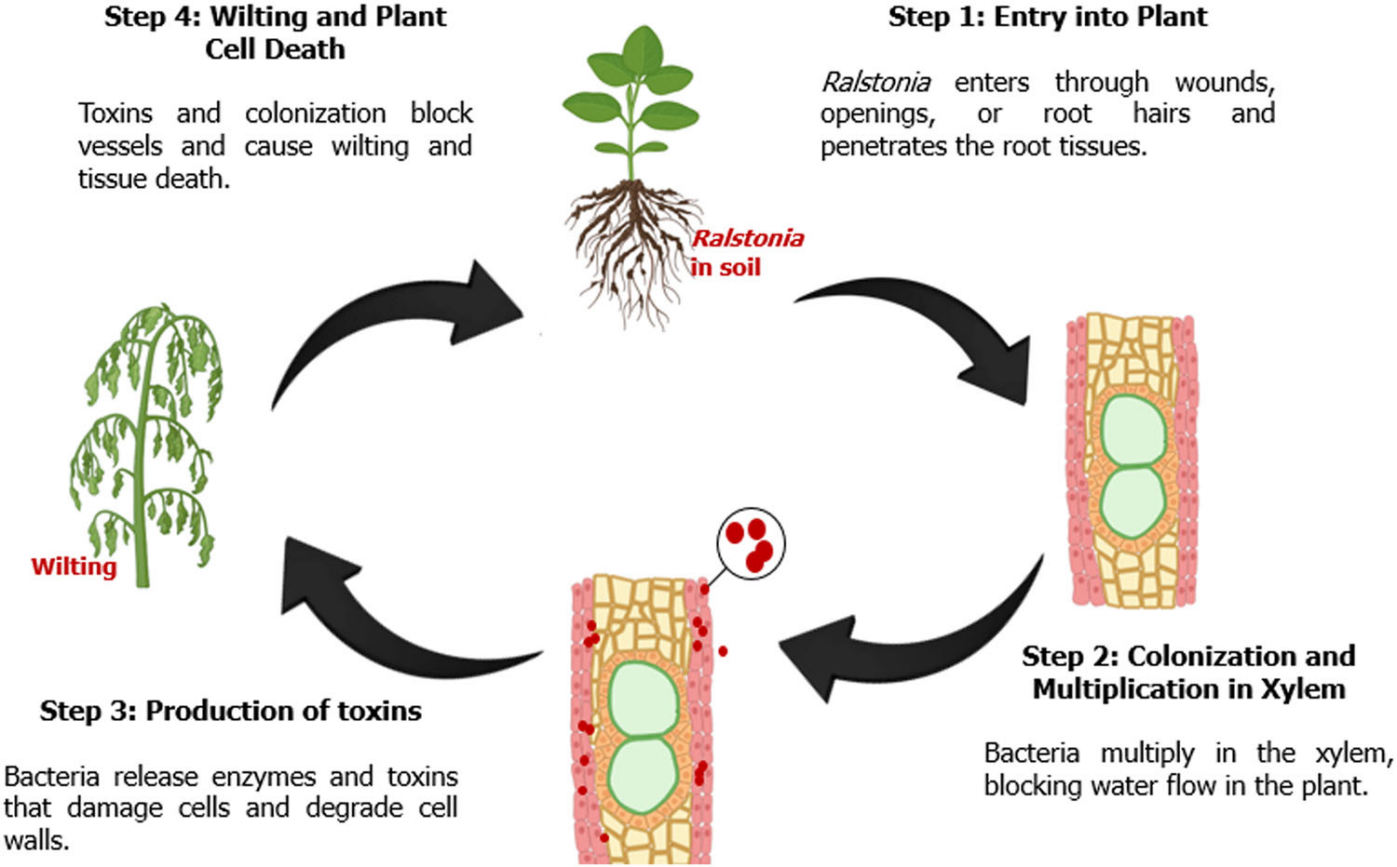
Pathogenicity mechanism of *Ralstonia solanacearum* in plants. The bacterium enters through roots, colonizes xylem vessels, produces cell wall‐degrading toxins, and results in wilting and tissue necrosis.

This pathogen invades the plant via wounds, roots, and other secondary root emerging points. Thereafter, the bacteria proliferate in the gaps between cortical cells, which eventually results in the plasmolysis of epidermal cells. Eventually, the bacterial infection will spread through the plant via xylem vessels (Figure 2). As the bacteria continue to multiply in the plant, they produce abundant exopolysaccharides (EPSs), which resemble loosely attached slime layers that will ultimately block the vessels. Subsequently, wilting symptoms will start to appear; as a result of impaired water conduction (Abdalla et al. 2021). Once the plants have wilted and started to dry up, the bacteria will then leave the root and will be released into the soil (Table 1) (Liu et al. 2022).

**Table 1:** The comparison between *Ralstonia solanacearum* and *Xanthomonas perforans*.

Feature	*Ralstonia solanacearum*	*Xanthomonas perforans*
Disease name	Bacterial wilt	Bacterial spot
Host range	Wide host range (tomato, pepper, potato, eggplant, tobacco, ginger, peanuts)	Narrow host range (tomato and pepper)
Main infection site	Root system and vascular tissue (xylem)	Fruit, leaf tissue and sometimes stems
Major symptoms of tomato	Wilting, vascular browning, stem ooze, collapse of plant without yellowing	Leaf spots with yellow halo, fruit lesions, defoliation, and necrosis
Type of pathogens	Soil‐borne	Seed‐borne
Mode of transmission	Soil, water, infected transplants/seedlings, contaminated equipment, wounds, and root contact	Water, infected seeds/transplants/seedlings, and contaminated equipment,
Virulence factors	Type III secretion system, cell wall‐degrading enzymes, extracellular polysaccharides, Type IV pilli, and flagella.	Type III secretion system, effectors, adhesins, exopolysaccharides, xanthan gum, and cell wall‐degrading enzymes.
Environmental preference	Warm, humid with high soil moisture (optimal 28°C–32°C), survives in soil and water	Warm and humid (optimal 25°C–30°C), survives epiphytically on leaves
Geographic distribution	Globally, prevalent in tropical and subtropical regions	Globally, common in warm, humid tomato‐growing areas

For the pathogen to progress across the different plant tissues, it relies on various virulence strategies. These include the production of exopolysaccharides, phytohormones, secretion of enzymes that play a role in degrading the cell wall, nutrient‐scavenging systems, detoxification, and motility. However, the main virulence factor is the Type III Secretion System (T3SS) (Su et al. 2021). The pathogenicity of *R. solanacearum* heavily depends on the T3SS secretion system, which injects Type III effectors (T3E) proteins into the plant cells. These effector proteins are referred to as *Ralstonia‐injected* proteins (Rips). To encourage bacterial invasion, they alter plant defenses while also interfering with host cell functions and plant immunity (Osemwegie et al. 2020).

Another key virulence is the secretion of EPS I, which is primarily responsible for the bacteria's ability to tolerate desiccation conditions in soil. When the pathogen passes through the host's physical barriers, it goes to the xylem and produces a large amount of EPS I. This leads to the xylem vessels getting clogged due to restricted water passage, which then results in wilting and eventually death. EPS I also binds to the wall and protects the bacterium from the plant defenses (Liu et al. 2022; Abdalla et al. 2021). *Ralstonia* also uses pili/fimbriae as a virulence factor. These are proteinaceous flexible filaments, which appear as hair‐like appendages on surfaces. The ones discovered in *Ralstonia* are Type IV pili (T4P), which are the most investigated. Although they serve many functions, they are primarily responsible for twitching motility, biofilm development, host cell adhesion, and DNA uptake (Y. Zhang et al. 2020). The virulence strategies described above are not the only ones used, although they are the most well‐known. Overall, *Ralstonia* has a wide host range, high genetic diversity, and a variety of virulence mechanisms that facilitate rapid infection and long‐term persistence. Its ability to thrive in a variety of environmental conditions and get past host defense highlights how threatening it is to tomato production. Therefore, creating focused and long‐lasting control strategies requires an understanding of the molecular underpinnings of its pathogenicity.

## 4 An Overview of *Xanthomonas* as a Phytopathogen

*Xanthomonas* is a rod‐shaped Gram‐negative bacterium that produces yellow pigments. It is ideal growth temperature ranges between 25°C and 30°C. The pathogen causes severe plant diseases in over 400 different plant hosts (Timilsina et al. 2020). Some hosts include major crops such as tomato, pepper, banana, citrus, cassava, cabbage, rice, wheat, and beans worldwide. *Xanthomonas* species are very host‐specific, allowing them to be divided into pathovars based on characteristic host range or tissue specialization. Furthermore, it is known to spend most of its life outside of its host plants, acting as an inoculum for subsequent infections of other plants (Alvarez‐Martinez et al. 2021).

**Figure 3:**
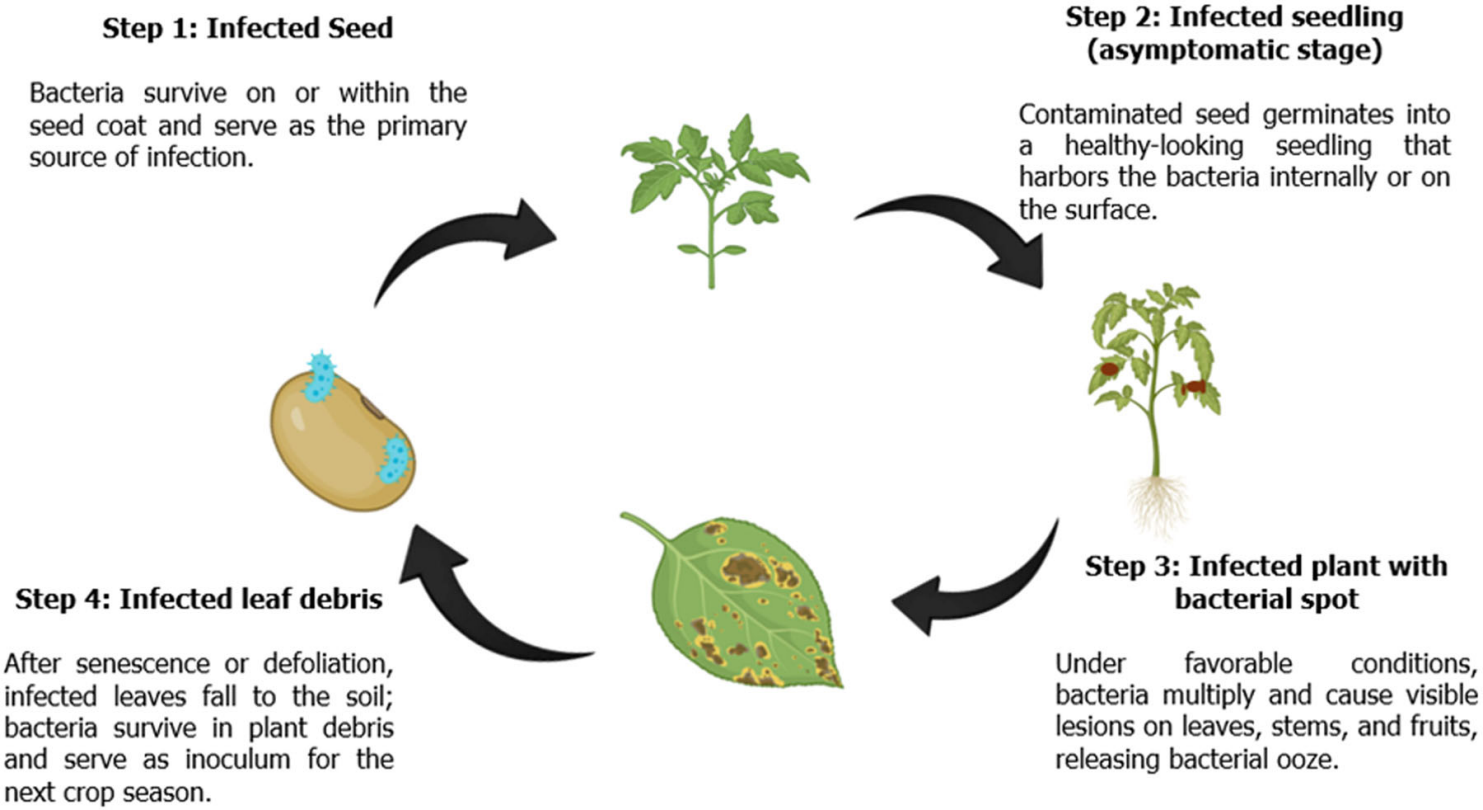
Pathogenicity mechanism of *Xanthomonas perforans*. This Disease cycle of *Xanthomonas perforans* shows seedborne infection, asymptomatic seedling stage, symptom expression on mature plants, and survival in infected debris.

*Xanthomonas* has two stages, which are epiphytic and endophytic. It is known to live in the lesions of fallen leaves or freely in the soil, acting as an inoculum for subsequent infection of the plant host. During the epiphytic stage, the bacteria will colonize the surface of the new host. Mechanisms used by the bacteria are adhesion proteins, polysaccharides, and type IV pili (Nakayinga et al. 2021). Afterwards, a biofilm will form, which will play a role in protecting the bacteria from hostile environmental conditions. Once within, the bacteria transition to the endophytic stage and begin to colonize the plant's internal structures. The endophytic stage begins when the bacteria enter the plant via stomata or wounds, and it eventually travels throughout the vascular system. During this process, the bacteria reach a high population density, re‐emerge on the plant surface, and are typically spread via wind or rain, and the infection cycle repeats when a new host is infected (Figure 3) (An et al. 2020).

For instance, with infected tomato plants, symptoms typically appear on aboveground plant parts such as stems, foliage, sepals, and fruits. It begins as little irregular water‐soaked lesions that eventually turn dry, black, and necrotic, and the lesion may occasionally form a faint halo. The lesions bond together in the last stage, and the plants become blighted and defoliated. As a result, the tissue in the middle collapses, revealing a shot hole. This disease can also infect the fruit, and an infected tomato begins with a small lesion that develops into a dark, scab‐like lesion with a yellow halo around it (Table 1) (Abrahamian et al. 2021a). Fruit lesions usually cause the fruit to become unmarketable and susceptible to opportunistic pathogen infections that lead to postharvest decays.

*Xanthomonas* species, as previously stated, use biofilm development as a virulence mechanism. It consists of lipopolysaccharides, exopolysaccharides, lipids, proteins, surfactants, and nucleic acid (Sabuquillo and Cubero 2021). The development of biofilm aids in the early stages of colonization and infection. It helps bacteria attach to surfaces and protects against harmful conditions like antibiotics and abiotic stress. Because of the biofilm, bacteria can withstand changes in temperature, pH, and other adverse environmental factors. Once the bacteria penetrate the host, the biofilm permits them to maintain optimal cell density in the host tissues (Sena‐Vélez et al. 2016).

The ability of *Xanthomonas* to cause disease is dependent on secretion systems (T1SS–T6SS), which play a role in how it interacts with host plants to suppress their immune response and facilitate nutrient acquisition and bacterial colonization (Alvarez‐Martinez et al. 2021). Each secretion system plays a specific, crucial role, and its significance will be discussed below. Type I secretion system has been reported to be conserved in all bacterial spot xanthomonads (Alvarez‐Martinez et al. 2021). It secretes protein that's involved in quorum‐sensing signaling, which plays a role in biofilm formation, which subsequently plays a role in adhesion, which facilitates the attachment of bacteria to the surfaces (Jibrin et al. 2022). They also play a role in transporting bacteriocins and toxins into the host plant (Green and Mecsas 2016).

Type II secretion systems are found in reference strains of the bacterial xanthomonads. It plays a role in enzymatic wall degradation. It secretes enzymes like proteases, cell wall lyase enzymes, xylanases, and lipases, which play a crucial role in the host cell wall degradation process (Korotkov and Sandkvist 2019; Dey and Raghuwanshi 2024). This system is crucial for the early stages of infection and penetration through natural openings or wounds. The type III secretion system plays a crucial role in pathogenesis and is conserved in all pathogenic xanthomonads. It transports the bacterial proteins called T3 effector (T3E) proteins into the host plant. These effector modify the host plant's cellular pathways, which benefits the pathogen by promoting multiplication, and also plays a role in suppressing the plant's immune response (Üstün et al. 2015). This system also secretes Transcription activator‐like effectors (TALE), which play a role in enhancing bacterial plasticity to host plants. They manipulate the plant gene expression system by binding to the plant's DNA and activating gene that enhances the plant's susceptibility to disease (Hutin et al. 2015; Perez‐Quintero and Szurek 2019). It also secretes XopJ effectors, which promote bacterial virulence by inhibiting the host cell proteasome, which results in delaying the defense response by suppressing the salicylic acid (Üstün et al. 2015).

Type IV secretion system plays a crucial role in competition by injecting antibacterial proteins into neighboring Gram‐negative bacteria, which results in rapid lysis upon contact, therefore eliminating competitive bacteria (Costa et al. 2024). Furthermore, it plays a role in horizontal gene transfer whereby genetic material is exchanged through conjugative plasmids, which subsequently helps with acquiring new traits directly involved in antibiotic resistance and novel virulence factors (Gordils‐Valentin et al. 2024). Type V secretion system plays a crucial role in transporting toxins, effectors, enzymes, and adhesins. These effectors aid in the pathogen being able to adhere to both host and non‐host and also biofilm formation (Dey and Raghuwanshi 2024). Type VI secretion system is a contact‐dependent system that plays in role in injecting toxic effectors into host plants as well as other bacteria, subsequently manipulating host cells as well as antagonizing other bacterial competitors residing in the same environmental niches (Ramamoorthy et al. 2024). *Xanthomonas* species have been shown to have type secretion systems in their genomes, although *X. perforans* does not have all six. T1SS, T2SS, T3SS, T5SS, and T6SS are present in most strains; however, T4SS is plasmid‐associated and confined to specific strains. As a result, not all secretion systems are conserved. throughout *X. perforans* genomes, and their presence is restricted (Alvarez‐Martinez et al. 2021).

In conclusion, *Xanthomonas* is a prime example of a highly versatile foliar pathogen whose success is driven by its varied armament of virulence, genetic adaptability, and capacity to endure in favorable environmental conditions. These traits not only make controlling it more difficult, but they also play a role in the frequent bacterial spot outbreaks that occur in tomato‐producing regions across the globe. Therefore, a thorough grasp of its pathogenic behavior and molecular variety is essential for directing the development of integrated and sustainable management strategies. In the section that follows, integrated methods for controlling tomato bacterial diseases are discussed, with a focus on the contribution of omics‐driven insights to long‐term disease control.

## 5 Integrated Management Strategies for Tomato Bacterial Disease

**Figure 4:**
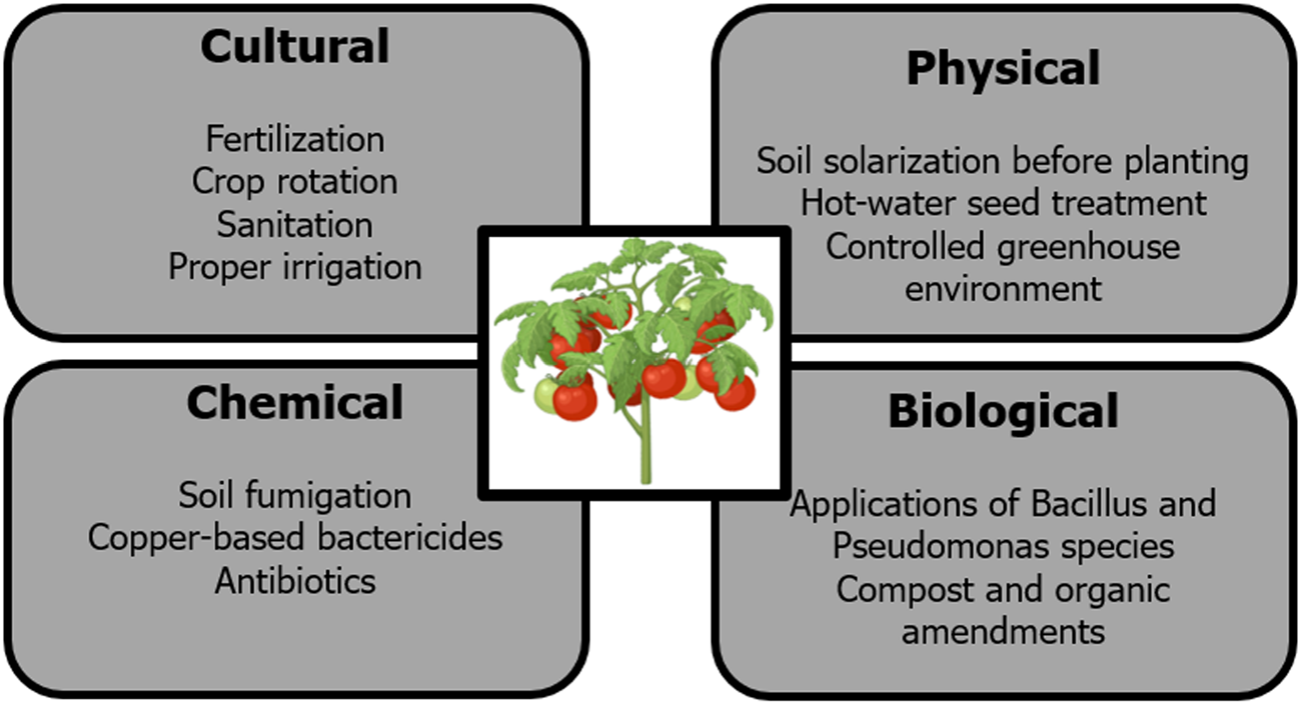
Disease management strategies for tomato bacterial diseases caused by phytopathogens. A combination of cultural, biological, chemical, and physical practices helps minimize pathogen populations, enhance plant resistance, and maintain sustainable crop production.

Management techniques continue to be a formidable task for farmers and plant pathologists since they demand a complete understanding of pathosystems to apply strategies at the proper time to target the pathogen population. Some of these management strategies include physical, cultural, chemical, and biological approaches (Figure 4) (Sundin et al. 2016). The physical approach consists of soil disinfection, hot water treatment, and soil solarization. Another technique is the cultural approach, which often tries to increase and promote crop yield and quality while limiting the impact of plant diseases (Panth et al. 2020). Crop rotation is a form of cultural approach, because when comparable crops are planted in the same location regularly, a specific pathogen's population may proliferate (Shah et al. 2021). This also helps reduce soil erosion. Another cultural approach is the utilization of fertilizers, particularly calcium‐rich ones. For example, plants with a high calcium content have lower pathogen populations and disease severity (Wang et al. 2023).

The most used approach is the utilization of chemicals. Farmers rely heavily on chemicals to control diseases in agriculture, and they continue to be a key component of Integrated Pest Management (IPM), as evidenced by the increasing use of fungicides, bactericides, and insecticides (Tudi et al. 2021; Pandit et al. 2022). Farmers worldwide are attempting to eliminate or limit the use of pesticides to manage plant diseases, and biological control is the most effective method. Biological approaches involve the utilization of natural agents, enemies, or bio‐based (animal, plant, or microbial) products to manage pests. This strategy is eco‐friendly, safe, sustainable, economically viable, and extremely specific to its objective. As previously stated, a better understanding of the plant, pathogen, and environmental factors is required (Pandit et al. 2022). Some of these biological control agents live in the soil and are known as plant growth‐promoting rhizobacteria (PGPR). These bacteria aid in both plant production and the battle against plant diseases (Santoyo et al. 2019).

Plants are protected from biotic and abiotic stress via direct and indirect mechanisms by these bacteria. Direct mechanisms include the acquisition of essential nutrients (nitrogen, phosphorus, iron, and so on) as well as the production of essential hormones. While indirect ways include the synthesis and production of toxic chemicals that will hinder the growth of plant pathogens, or being antagonistic towards phytopathogens by competing for critical nutrients (Ahmed et al. 2022).

### 5.1 Pathogen‐Specific Integrated Management Strategies

Bacterial wilt induced by *Ralstonia* is difficult to control after it has established itself in the soil. This pathogen has a wide host range and is capable of persisting in the soil for long periods, even in extreme environmental conditions. It also has a high diversity, which adds to its difficulty in management (Mamphogoro et al. 2020). Its ability to grow endophytically, live in soil, especially the deeper layers, migrate with water, and interact with weeds makes control difficult (Ye et al. 2022). Therefore, although there are methods put in place to manage the disease, there are limitations. Due to its ability to generate quick and lethal wilting symptoms in host plants, it is considered the second most important bacterial phytopathogen and the most destructive disease found to date (Yuliar et al. 2015).

Numerous approaches, such as physical, chemical, cultural, and biological control, have been put in place to try and manage bacterial wilt of plants by *Ralstonia*. For instance, with a physical approach, the soil is disinfected, and hot water treatment, and soil solarization (Benti 2023). In order to raise the soil temperature before planting, hot water treatment involves adding hot water to the soil at a temperature between 70°C and 90°C. The high temperature kills pathogens, pests, and weed seeds. Although this treatment does not fix all the problems, it is environmentally friendly because it does not disrupt the soil microbiota. For example, bacteria that are heat‐resistant and form spores can live and replenish the soil once the soil has cooled down, which increases resistance to plant diseases (Mamphogoro et al. 2020).

With solarization, the heat that is trapped in the soil helps eliminate harmful organisms and removes weeds without leaving any toxic residues behind. This is achieved by covering the soil with plastic during high temperatures, and the captured heat subsequently suppresses the pathogen population in the soil (Wang et al. 2023). This method also eradicates insects and nematodes. The primary drawback is the possibility that some advantageous bacteria would suffer the same fate as their detrimental counterparts (Mamphogoro et al. 2020).

Another approach is a cultural approach, which includes using resistant cultivars, crop rotation, grafting, and soil amendment to increase crop yield and quality and to minimize the impact of pathogens. The best and greenest approach is to use resistance cultivars that are extremely resistant to bacterial wilt (Vargas et al. 2023). Some resistant cultivars that have been used and were successful in resisting *Ralstonia* are crops like eggplant, peanut, pepper, and potato. Crop rotation is a technique used to minimize diseases caused by soil‐borne pathogens, improve soil quality and health, and combat pathogens by planting various crops on the same plot of land in succession (Ajilogba and Babalola 2013). When similar crops are planted on the plot of land, it encourages pathogens to proliferate in the soil.

Soil amendment is the process of adding organic matter to topsoil to improve soil qualities (chemical, biological, and physical) as well as overall plant development and health (Palansooriya et al. 2020). Soil additives frequently contain biologically active compounds like growth regulators, toxins, and vitamins, which can affect microorganisms directly or indirectly. These compounds can then stimulate microorganisms with pathogen‐specific antagonistic activity. For example, silicon fertilizers and sugarcane bagasse have shown to reduce bacterial wilt incidence; applying farm manure compost or coco peat increased tomato fruit yield while also reducing bacterial wilt; and increasing calcium concentration in the soil was shown to reduce *R. solanacearum* in tomato stems (Wang et al. 2022). Grafting is another cultural practice that involves connecting two plants so that they can grow as one. The upper component of the graft (the scion) becomes the plant's top, while the lower piece (the understock) becomes the root system or a section of the trunk (Rasool et al. 2020). The scion will be the desired cultivar, whereas the rootstock will be resistant. The primary goal of this strategy is to develop crops that are resistant to soil‐borne diseases.

The chemical technique is the most often utilized way for controlling infections, pests, and weeds. Benomyl, carbendazim, propiconazole, and flubendazole are among the compounds used. Pesticides used to control bacterial wilt include fumigants (metam sodium, 1,3‐dichloropropene, and chloropicrin), algicides (3‐[3‐indolyl] butanoic acid), and plant activators (validoxylamine and validamycin A). Chemical techniques have been employed to combat bacterial wilt for many years; however, due to the pathogen's intricacy, no single technique is effective (Nazarov et al. 2020). Pesticides are the most efficient technique for controlling bacterial wilt; however, improper application has resulted in the toxins staying in the environment for years. Subsequently, they become contaminants in the soil and groundwater, which can be toxic to humans (Rajmohan et al. 2020).

A preferable alternative is to use biological control agents, as this strategy not only inhibits infections but also enhances general plant health (Adhikari et al. 2020). Several studies have demonstrated the efficacy of certain biological control agents that suppress the establishment of *R. solanacearum*, *B. subtilis,* and *B. thuringiensis* secrete antimicrobials, enzymes that degrade bacterial cell walls, and inhibit bacterial growth, and lipopeptides (Karačić et al. 2024). Furthermore, some strains also induce systemic resistance (ISR) in hosts, which puts the plant's immunity on alert, therefore reducing vascular colonization by the pathogen (Morales et al. 2022).

**Figure 5:**
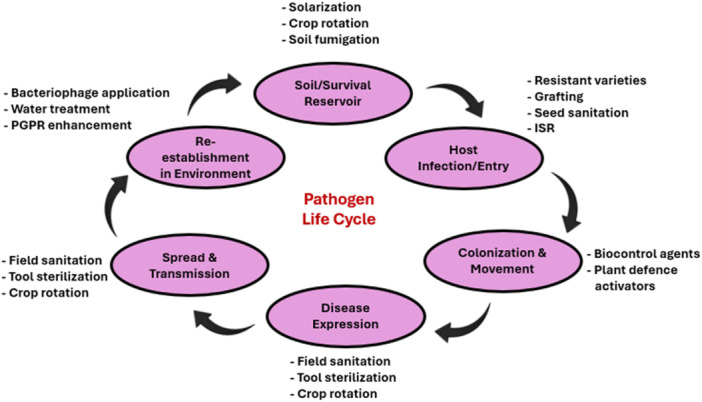
Illustration of disease management strategies targeting different stages of the pathogen life cycle. Highlighting the importance of integrating multiple methods for effective disease control.

*Pseudomonas fluorescens* has been reported to play a role in competition by colonizing the rhizosphere, and also antagonizes the pathogen by secreting siderophore metabolites (Sah et al. 2021). Furthermore, some strains are reported to attenuate the pathogen's virulence by interfering with quorum sensing (Gutiérrez‐Pacheco et al. 2019). The effectiveness of these BCAs is related to direct antagonism, competitive exclusion, and inducing the host's resistance. The effectiveness of disease management strategies often depends on their timing, as different methods act on specific stages of the disease development cycle (Figure 5).

The management of bacterial spots induced by *Xanthomonas* species includes cultural, chemical, and biological control measures. Studies have revealed that it is vitally critical to apply proper sanitation procedures to the seeds before planting because *Xanthomonas* is known to live and grow on tomato seeds (Abrahamian et al. 2021a). Some of the cultural treatments include cutting lower leaves and tying plants to increase airflow, as well as pathogen‐free certified seed and disease‐free transplants. Weeds should also be eradicated. Crop rotation is one of the most popular ways, and it helps to prevent carry‐over pathogens from the previous crop (Soto‐Caro et al. 2023).

In the chemical approach, antibiotics are used to control bacterial spot disease. Streptomycin has been used for years; however, the pathogen has developed resistance to it. The antibiotic streptomycin, which was initially identified in 1944, is generated by the bacteria *Streptomyces griseus*. Mainly used to prevent *Xanthomonas* from synthesizing proteins, this antibiotic exhibits a broad spectrum of activity. Other substances employed include copper and copper‐based compounds (Sundin and Wang 2018). Copper and copper‐based compounds have also been employed in agriculture to treat various phytopathogens, including bacteria. Copper is an oligo‐element that is required for physiological functions. This metal is a prominent component of fungicide and bactericide formulations worldwide (Tamm et al. 2022). The downside of copper treatment is that, because it is sprayed on the foliar, it is not absorbed by the plant and remains on the surfaces as residue. Finally, the metal reaches the soil through the wind or is rinsed by rain or irrigation. Because copper is not degradable, it will persist in the soil as a contaminant in the ecosystem, causing bioaccumulation and toxicity (La Torre et al. 2018). Furthermore, their widespread use has resulted in resistance among the *Xanthomonas* population. Acibenzolar‐S‐methyl (ASM) is an alternative to copper and streptomycin (Abrahamian et al. 2021a).

A field study was undertaken in Serbia to investigate the effectiveness of several ways that might be included in an integrated management of bacterial spots of pepper caused by *Xanthomonas euvesicatoria*. Although measures are in place to control the disease, they are insufficient, necessitating the development of alternative or combined solutions. In their study, they investigated the efficacy of copper‐based compounds in combination with or without mancozeb, antibiotics (streptomycin sulfate and kasugamycin), systematic acquired resistance (SAR) induced (ASM), microbial fertilizer (Slavol), and biocontrol agents (bacteriophage strain KФ1, Bacillus subtilis AAac, and QST 713). When examined separately, all treatments, except microbiological fertilizer and antagonistic strains, were found to have considerably lower illness intensity. However, the combination of copper hydroxide, bacteriophages, and ASM was the most effective treatment, which reduced the disease severity by 96%–98%. These findings imply that the combination may be the most effective way to treat bacterial spot‐on pepper (Šević et al. 2019).

Another study was done to assess the efficacy of antibiotics and fungi toxicants (single or combined) against *Xanthomonas oryzae* pv. *Oryza* which causes bacterial leaf blight in rice. Streptocycline, Streptomycin, Streptomycin sulfate, Plantomycin, Tetracycline hydrochloride, and Oxytetracycline hydrochloride were the six antibiotics utilized. Tetracycline hydrochloride was shown to have the largest zones of inhibition, followed by streptomycin. Five fungi toxicants were evaluated, including copper hydroxide, carbendazim, validamycin, and propiconazole. Validamycin had the largest zone of inhibition, followed by copper oxychloride. Various antibiotics and fungicides were tested in combination treatments. Antibiotics included streptocycline, streptomycin, streptomycin sulfate, plantomycin, tetracycline hydrochloride, and oxytetracycline hydrochloride. Fungitoxicants included copper oxychloride, copper hydroxide, and carbendazim. The treatment with Streptocycline and Carbendazim, Tetracycline hydrochloride and Copper oxychloride, and Streptomycin sulfate and Copper hydroxide yielded the largest zone of inhibition. This study showed that various antibiotics can be successful when used on their own, as well as when paired with fungi toxicants (Deep et al. 2020).

*Pseudomonas* species are hostile to *Xanthomonas* phytopathogens, and new species are being discovered all the time. A study was done to evaluate *P. oryziphila* 1257s biological control efficiency towards *X. oryzae* pv. *oryzicola* (*Xoc)* RS105, which is the causal agent of bacterial leaf streak (BLS) in rice. Rice leaves from a susceptible cultivar were sprayed with *Pseudomonas* first, followed by *Xanthomonas* inoculation. It was discovered that *P. oryziphila* 1257 was antagonistic towards both pathovars. The findings indicated that the *P. oryziphila* 1257 strain may be a suitable biological control agent for BLS (Yang et al. 2021).

*Bacillus* is a well‐studied species for biological control. It is well‐recognized for producing antibacterial compounds, which have led to its classification as a biological control agent. A study was done to examine the potential of *Bacillus* spp. as a biological control agent. This was done in Taiwan, and they were testing its efficacy against *Xanthomonas citri* subsp. *citri* (*Xac*), which causes citrus canker. These trains were made with potting mix, dirt, and organic compost. There were 326 strains to be retrieved, and seven were chosen to further examine their potential antagonistic actions. Further analysis revealed that these strains may be intricately linked to *Bacillus subtilis*. Two strains were further investigated, and it was reported that strains TKS1‐1 (*B. subtilis*) and WG6‐14 (*B. amyloliquefaciens*) lowered symptom development, which was based on a reduction in colonization and as well as biofilm formation by the bacterial cells (Herbert et al. 2022). To highlight the similarities and differences in managing these two pathogens, the following table compares disease management approaches for *Ralstonia* and *Xanthomonas* (Table 2).

**Table 2:** Comparison of disease management approaches for *Ralstonia solanacearum* and *Xanthomonas perforans*.

Approach	Application	Mode of action	Pathogen
Cultural	Crop rotation	Reduces soil inoculum and breaks the disease cycle	Both
	Grafting	Limits pathogen entry and improves plant tolerance	Both
	Utilize pathogen free seeds/transplants	Prevents initial introduction	Both
	Use sterile equipment	Reduces cross‐contamination	Both
	Control weeds and volunteer plants	Eliminates alternative hosts	Both
	Application of fertilizer	Enhances plant vigor and improves immunity	Both
	Utilize resistant cultivars	Limits pathogen entry and improves plant tolerance	Both
	Avoid overhead irrigation	Reduces leaf wetness and the spread of foliar disease	*X. perforans*
	Improve field drainage	Minimizes waterlogging and pathogen proliferation	*R. solanacearum*
	Soil solarization	Reduces soil‐borne inoculum	*R. solanacearum*
Chemical	Copper‐based compounds	Disrupts bacterial cell membranes and enzymatic systems	*X. perforans*
	Streptomycin antibiotic	Inhibits bacterial protein synthesis	*X. perforans*
	Plant defense activator (acibenzolar‐S‐menthyl)	Activates systemic acquired resistance pathways in plants	*X. perforans*
	Soil fumigation	Disrupts survival of pathogen	*R. solanacearum*
Biological	*Bacillus subtilis*	Produces antimicrobials and competes for nutrients	Both
	*Pseudomonas fluorescens*	Produces antimicrobials and siderophores	Both
	*Streptomyces spp*.	Produces cell wall‐degrading enzymes	*X. perforans*
	*Bacillus thuringiensis*	Produces antimicrobials and disrupts cell membranes	*R. solanacearum*
	Bacteriophages	Lyse bacterial cells	Both
	PGPR	Indirectly suppress disease by improving immunity and increasing nutrient uptake	Both

### 5.2 Limitations and Emerging Challenges in Integrated Disease Management

Despite advancements in the development of integrated management strategies against *Ralstonia solanacearum* and *Xanthomonas perforans*, various challenges prevent common field‐level effectiveness. These include the development of resistance, regulatory restrictions, and effects of climate change, all of which have an impact on the dynamics of the pathogen and the effectiveness of management (Chachar et al. 2025; Adhikari et al. 2020; Bhandari et al. 2023). For instance, the long‐lasting reliance on chemical bactericides has contributed to pathogens developing antimicrobial resistance. The efficiency of chemical bactericides, particularly those based on copper and antibiotics, has been compromised by the advent of resistant strains of *Xanthomonas (*Kaur et al. 2024; Carvalho et al. 2019). *Xanthomonas* strains have developed copper‐based resistance by encoding plasmids with CUR genes (Kaur et al. 2024; Marin et al. 2019). *Ralstonia solanacearum* can overcome host resistance genes through horizontal gene transfer and mutation due to its genetic plasticity (Chen et al. 2022; Coupat‐Goutaland et al. 2011). Breeding for long‐lasting resistance is made more difficult by the diversity of the *Ralstonia solanacearum* species complex, which includes several phylotypes and races (Okiro et al. 2022; Jimenez Madrid et al. 2019). Furthermore, persistent use of a single biocontrol strain without diversity or rotation may eventually result in decreased efficiency because of pathogen adaptation or microbial competition (Niu et al. 2020).

This development in resistance is observed for other antibiotic treatments where streptomycin and oxytetracycline are extensively used, this results in reduced pathogen sensitivity and treatment failures (Miller et al. 2022; Herbert et al. 2022). Antimicrobial resistance affects the efficacy of chemical approaches and highlights the importance of developing sustainable integrated strategies (Batuman et al. 2024). Incorporating diverse management strategies like reducing pathogen inoculum with cultural practices, resistant cultivars, and biological control agents that use different modes of action (He et al. 2021; Cucu et al. 2025). In order to improve disease management strategies, it is crucial to monitor antimicrobial resistance to develop sustainable and strengthen integrated management approaches against tomato bacterial diseases (Meenakshi Raman et al. 2021). Omic‐based approach is the tool that can help with this improvement by helping to identify resistance genes, which will help in guiding which is the best approach for a specific phytopathogen. This approach will further aid in investigating potential biological control candidates (Wijayawardene et al. 2023; Gudeta et al. 2024; Kimotho and Maina 2024).

The commercial licensing and widespread use of biological control agents (BCAs) are delayed by regional variations in regulatory frameworks. Infrastructure for formulation, quality control, and farmer education on the safe application of BCA is lacking in many developing countries (Mawcha et al. 2025). Additionally, policy harmonization and extension support, which are often lacking, are necessary for the integrated use of biocontrols with suitable pesticides or fertilizers. The implementation of eco‐friendly management techniques in the fields is obstructed by these gaps (Jaiswal et al. 2022; Pratissoli 2025). Both pathogen behavior and host vulnerability are significantly impacted by climate fluctuation (Kumar and Mukhopadhyay 2025; Lahlali et al. 2024). The growth and spread of *R. solanacearum*, a soilborne pathogen that thrives in warm, high‐moisture soils, are facilitated by warmer temperatures (Wang et al. 2023). Similarly, excessive humidity and temperatures that promote bacterial growth and dissemination intensify *X. perforans* epidemics (Abrahamian et al. 2021b). Additionally, the geographic distribution of many diseases may shift due to climate change, perhaps prolonging disease seasons or introducing them to new regions (Singh et al. 2023). As a result, previously resistant cultivars may become susceptible to new environmental stressors. Therefore, creating climate‐resilient management systems, such as adaptive resistance breeding and predictive modeling, is crucial for long‐term disease control (Rivero et al. 2022; González Guzmán et al. 2022; Razzaq et al. 2021).

Despite the experimental testing of several integrated techniques, there is still limited field validation and low farmer acceptance (Tratwal et al. 2025). This is frequently brought on by insufficient knowledge sharing, financial limitations, or ignorance of integrated pest management principles (Tiemann and Douxchamps 2023). Encouraging participatory approaches, farmer‐led demonstrations, and cross‐sectoral collaboration among researchers and policymakers could bridge these gaps (Prajapati et al. 2025; Adamsone‐Fiskovica and Grivins 2022).

## 6 OMICS and CRISPR Tool for Unraveling Host–Pathogen Interactions in *Xanthomonas* and *Ralstonia* Pathosystems and Their Implications for Disease Management

Although traditional disease management practices (cultural, chemical, and biological) offer various levels of control, the omics approach provides deeper insight to improve and refine these strategies. The ecology of plant diseases is inherently interdisciplinary, drawing on microbial ecology, plant physiology, epidemiology, and genetics to understand how and why plants become infected (Crandall et al. 2020). Historically, plant disease studies adopted a reductionist lens, isolating components rather than examining the complex ecological interactions among microbial communities, host plants, and the environment. However, advances in technology—particularly omics‐based approaches—now allow a more holistic view of disease dynamics (N. Zhang et al. 2020).

Omics technologies such as genomics, transcriptomics, and metabolomics have allowed researchers to conduct in‐depth studies of plant‐microbe interactions (Jain et al. 2024). Genomics allows researchers to identify genes associated with resistance, pathogenicity, and adaptation in plants and pathogens (Xu and Wang 2019). Transcriptomics investigates gene expression under specific conditions in order to learn how pathogens regulate genes during infection and how plants generally respond at the molecular level to infection (Xia et al. 2017). Metabolomics, on the other hand, analyses small molecules known as metabolites, providing insight into how biochemical pathways function during pathogen attack and plant defense (Lu and Xia 2025). In order to demonstrate the application of these omics technologies, the following section highlights a few studies that have investigated plant–pathogen interactions involving *Xanthomonas* and *Ralstonia*.

A genomics study looked into the role played by a particular gene within the type VI secretion system cluster of *X. perforans* during early, asymptomatic stages of the infection. The targeted gene mutation and the comparative phenotypic analysis revealed that the particular gene played a role in epiphytic fitness and reduced aggressiveness, which are crucial for the survival of the pathogen. Furthermore, bacterial growth and disease severity were enhanced when that gene was silenced. This suggests that this gene is a potential target for designing disease control strategies in the early stages (Liyanapathiranage et al. 2022).

Transcriptomics studies have further expanded our understanding of host‐pathogen interactions. For instance, a transcriptomics study was conducted, and comparative RNA‐seq analysis between tomato roots infected with wild‐type hrpB‐mutant *R. solanacearum* revealed various commonly dysregulated genes, and this included kinase WALK20, which was suppressed during infection. Therefore, transcriptomics uncovered the mechanisms used by the pathogen to manipulate the host's virulence factors (Liu et al. 2023).

In another transcriptomics study, genome‐wide gene expression analysis was conducted on the roots of a resistant as well as a susceptible tomato cultivar, at various time points after being inoculated with *R. solanacearum* strain. It was reported that the defense pathways in the resistant cultivars were activated much rapidly and intensely than the susceptible roots. Other findings included auxin and transport pathways were suppressed in the resistant cultivar, which aids in enabling the plant defense to be activated. Therefore, this study provided insight into how root tissue contributes to bacterial resistance, which will help to identify target pathways for breeding and disease management strategies (French et al. 2018).

In this particular study, genomics and transcriptomics played a crucial role in uncovering the molecular basis of *R. solanacearum* infection in pepper. Firstly, various genes and about 84 type III effectors linked to virulence were detected in the strain through Whole‐genome sequencing. Lastly, transcriptomics of the profiling of the infected pepper plants identified thousands of differentially expressed genes, which suppressed photosynthesis, enhanced ethylene biosynthesis, and others that were linked to weakening cell wall defense. Overall, the integration of the omics approaches showed insight in both host susceptibility and pathogen virulence, which shows potential target areas for plant breeding and disease control (Jain et al. 2024; Ramlal et al. 2023).

Overall, omics technologies are becoming crucial in plant disease ecology, particularly in unraveling the mechanisms of pathogenesis and resistance in host–pathogen systems involving *Xanthomonas* and *Ralstonia*. By integrating omics tools, this facilitates in development of targeted and sustainable disease management strategies. While omics offer a deeper understanding of the host‐pathogen complex, CRISPR plays a role in enabling precise manipulation of genes that play a role in pathogenicity or resistance (Echavarria Galindo and Lai 2025). Advances in CRISPR‐Cas genome editing have transformed research on plant‐microbe interactions. The CRISPR‐Cas system, which was first discovered as an adaptive immunity mechanism in bacteria, allows for precise, targeted DNA sequence modifications (Park et al. 2025). In plant pathology, this technology has proven to be effective tool for functional genomics and disease management (Gong et al. 2025; Chen et al. 2024). Researchers can use CRISPR‐Cas to investigate the molecular basis of host‐pathogen interactions, knocking out or altering genes implicated in pathogen virulence, host vulnerability, and defense responses (Banerjee 2025; Jothi et al. 2021).

CRISPR‐Cas9‐mediated editing of susceptibility (S) genes has become a particularly effective tactic in tomatoes. As an illustration of the possibility of CRISPR to produce long‐lasting, multi‐pathogen resistance without introducing foreign DNA, deletion of the SlDMR6‐1 gene provided broad‐spectrum resistance to bacterial, fungal, and oomycete pathogens (Thomazella et al. 2021). Similar to this, CRISPR has accelerated the functional validation of candidate genes found by omics techniques, such as those involved in hormone signaling or pathogen recognition, allowing for accurate assessment of their roles in disease susceptibility or resistance. CRISPR has successfully edited the CaMLO2 gene in pepper (*Capsicum* spp.), increasing resistance to powdery mildew and demonstrating the technology's versatility beyond tomato (Park and Kim 2023). By directly assessing gene function, this research highlights CRISPR's ability to supplement omics‐driven discoveries and close the gap between genome‐wide data and workable disease control techniques.

Its influence is further increased by recent developments in multiplexed and high‐throughput CRISPR techniques. For instance, the development of large‐scale CRISPR libraries in tomatoes makes it possible to target several gene families at once, which speeds up the screening of pathogen‐responsive genes and the discovery of new resistance mechanisms (Berman et al. 2025). Additionally, base and prime editing methods offer additional ways to create resistant features while reducing off‐target impacts by allowing for precise alterations without creating double‐stranded breaks.

Overall, by facilitating the functional validation of pathogen‐responsive genes, speeding up resistance breeding, and providing creative, sustainable substitutes for chemical disease management, CRISPR‐Cas technologies have had a significant impact on plant pathology. CRISPR offers a powerful framework for figuring out intricate host‐pathogen interactions and creating focused, long‐lasting control techniques in tomato, pepper, and other crops when combined with omics insights.

## 7 Conclusion

Bacterial diseases of tomatoes produce devastating diseases in the fields and greenhouses. As a result, farmers rely largely on chemical management measures to manage diseases while also promoting plant health and growth. Different solutions have been utilized; however, there are limitations, and farmers are attempting to transition away from chemical use. Biological control agents, on the other hand, are recognized as the best alternative strategy for reducing pathogenic bacteria, promoting growth, and increasing stress tolerance. Biological control agents have been investigated over time, but further research is required. Omics technologies help to gain a better knowledge of host‐plant interactions, allowing for the development of new and improved bacterial infections in plants.

## Author Contributions

All authors contributed equally.

## Ethics Statement

The current work follows the ethical requirements for publication. It does not involve human subjects, animal experiments, or any data collected from social media platforms.

## Consent

All authors approved the manuscript for publication.

## Conflicts of Interest

The authors declare no conflicts of interest.
